# Cognitive Strategies: Moderating the Relationship between Executive Functions and Daily Functioning

**DOI:** 10.3390/ijerph192416845

**Published:** 2022-12-15

**Authors:** Yael Fogel

**Affiliations:** Department of Occupational Therapy, Ariel University, Ariel 40700, Israel; yaelfo@ariel.ac.il; Tel.: +972-526162455

**Keywords:** cognitive strategies, executive function, daily performance, cognitive load, self regulation

## Abstract

Whereas prior studies have addressed relationships between cognitive strategies and learning and achievement, very few dealt with their connection to daily functioning. This study examines the moderation effect of the frequency of compensatory cognitive strategy use within that relationship among university students. A sample of 336 students (18–36 years; 180 women, 156 men) answered the Dysexecutive Questionnaire (DEX; executive function components), Time Organization and Participation Scale (TOPS; daily functioning), and Compensatory Cognitive Strategies Scale (CCSS; strategy use). The results showed significant correlations between the DEX and TOPS for three CCSS levels (−1.0, −1.0 to 1.0, and 1.0 SD from average); the higher the frequency of cognitive strategy use, the stronger the association between the DEX and TOPS. The findings suggest that more frequently use of cognitive strategies can strengthen efficient daily functioning.

## 1. Introduction

Successful academic performance requires cognitive strategies, such as setting reminders, minimizing distractions, planning and prioritizing a “to-do” list, notetaking, and developing new study strategies to remember important material. For students with or without cognitive weaknesses or impairments, compensatory cognitive strategy use can help successful academic performance during postsecondary studies [1].

*Strategies* are a part of typical learning and performance [2] and inherent in everyone’s daily occupational performance [3]. They can be described as “mind tools” that assist in acquiring new information, enhancing understanding, monitoring performance, and coping with cognitively challenging or complex situations [4]. With the help of strategies, people can take in and integrate information more deeply and increase the efficient use and allocation of cognitive resources [4].

According to Toglia’s [3] dynamic interactional model, appropriate strategy selection increases the efficient use of information-processing resources, and such strategic behavior reflects intact executive functions (EF). All strategies enhance information processing and allow tasks to be completed with less effort and more efficiency. *Strategy generation* occurs when a person faces a complex or novel situation or task. A task is considered complex if it takes place over long periods, requires the choice of a solution from among many possibilities, or occurs in a dynamic, real-world environment where the examiner does not control numerous environmental variables [5]. The suggested (or generated) cognitive strategies can be combined, adjusted, or modified to help cope with cognitive challenges and support effective functioning [6].

*Cognitive strategies* are a particular class of strategies involved in all activities requiring EF. Executive functions encompass a diverse array of goal-directed functions, such as problem-solving, cognitive flexibility, planning, monitoring, decision-making, working memory, and inhibitory control [7]. The need for EFs to complete the activity depends on the task’s complexity and the degree of familiarity and structure provided [8]. Components of EF can be observed when people carry out daily life activities that require time multitasking, goal formulation, or specific activity sequences for successful completion; face changing rules and must be flexible or change strategies; or must ignore competing stimuli to maintain goal-directed activities [9,10]. 

Many researchers have noted differences among students in cognition and the application of cognitive strategies. Proctor [11] pointed out a significant relationship of students’ cognitive-strategy ability (especially learning information processing speed, comprehension and understanding power, and working memory) with learning and academic achievement, especially in mathematics. Ganbari-Taleb et al. [12] showed the effectiveness of cognitive strategies in improving academic functioning and positive attitudes toward learning. 

One reason for students’ disabilities in the educational process relates to weak awareness and use of cognitive strategies. The evidence has suggested that a learner can improve performance by modifying strategies, and unlike poor learners, good learners spontaneously apply deep processing strategies [2]. The ability to organize and execute daily life tasks within time constitutes an essential component of efficiency and may influence a person’s participation and success in life events [13]. 

Studies about the need and role of cognitive strategies and their relationships to EF and daily functioning were conducted mainly among populations with learning or attention difficulties [14] and traumatic brain injury [5]. Despite demonstrated associations between EF and daily performance, little of the literature addresses the role of cognitive strategies in moderating these relationships [15,16]. 

**Figure 1:**
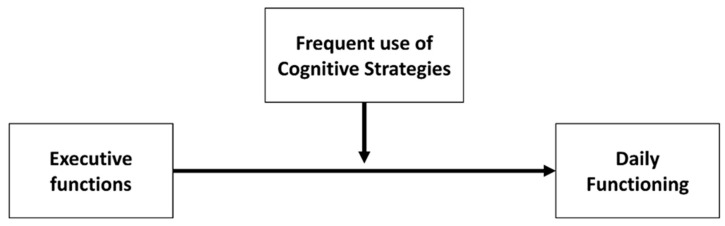
Moderating effects of the use of cognitive strategies on the relationship between EF and daily functioning.

Therefore, this study’s main objectives were to characterize the frequency of cognitive strategies among students and explore the strategies’ contributions to the relationship between EF and daily functioning (Figure 1). The main hypothesis is that a high frequency of cognitive strategy use will strengthen the relationship.

## 2. Materials and Methods

### 2.1. Participants

Using G*Power software guidelines, the author determined a minimum sample size of 169 participants considering the medium effect size of 0.15, power = 95, and α = 0.05 [17]. A total of 336 students (180 women, 156 men) aged 18 to 40 years from universities across Israel invited to participated in the study. Inclusion criteria were (1) students who could read and understand the Hebrew language (the questionnaires were presented to the participants in Hebrew), (2) students enrolled in Israeli universities, and (3) between the ages of 18 and 40 years. Students with disabilities (motor, mental, cognitive, communication, neurodevelopmental, etc.) were asked to report them when completing the demographic questionnaire.

### 2.2. Procedure

Following ethics committee approval, participants were recruited via advertisements to local online students’ groups and social media. Interested participants who met the inclusion criteria signed online consent forms and then were provided links to the online questionnaires (Demographic Questionnaire, Compensatory Cognitive Strategies Scale [CCSS], Dysexecutive Questionnaire [DEX], and Time Organisation and Participation Scale [TOPS]).

### 2.3. Measures

#### 2.3.1. Demographic Questionnaire

The author developed a short demographic questionnaire for this study. It included characteristics such as age, gender, family status, field of study at the university, grade range in the last year, whether employed while studying, and previous first degree. It also asked about motor (e.g., cerebral palsy, motor clumsiness) or other physical disabilities, mental disabilities (anxiety, depression, sleep disorders, mental disorders such as schizophrenia), cognitive disability (after a stroke or head injury, etc.), communication disability with some language difficulty (lack of fluency, dyspraxia), autistic spectrum disorder, or neurodevelopmental disorder (e.g., learning disability, attention deficit disorder, or motor clumsiness).

#### 2.3.2. Compensatory Cognitive Strategies Scale

The CCSS [18] is a 20-item questionnaire on how frequently participants use compensatory cognitive strategies in their everyday lives. It queries strategies related to processing speed and attention, memory, and EF. Participants are instructed, “Please rate how frequently you use each strategy to complete cognitive tasks (e.g., those involving thinking, attention, memory) in your daily life.” They rate each item’s frequency of use on a 5-point Likert-type scale of 0 (*never*), 1 (*rarely*), 2 (*sometimes*), 3 (*often*), and 4 (*all the time*). Higher scores mean the strategy is used with more frequency; lower scores mean less frequency.

For patients with multiple sclerosis, previous research has shown acceptable internal consistency for the CCSS total score (α = 0.90) and evidence of convergent and discriminant validity [18]. With the CCSS author’s permission, the questionnaire was translated into Hebrew for this study. The questionnaire’s internal reliability for this study was 0.81

#### 2.3.3. Dysexecutive Questionnaire

The DEX [19] examines individuals’ functional management levels in coping with day-to-day functioning. The questionnaire is part of the Behavioral Assessment of the Dysexecutive Syndrome assessment but stands on its own. It contains two versions, one for family members and one for self-reports. Only the self-report was used in this study. 

The DEX includes 20 statements graded on a 5-point Likert scale from 0 (*never*) to 4 (*very often*) and divided into four areas: emotion/personality, motivation, behavior, and cognition. The final score is the mean of all 20 items; higher scores indicate a higher incidence of EF challenges in daily life. The DEX is sensitive and ecologically valid for identifying symptoms that characterize executive dysfunction in healthy adults and in various neurological and psychiatric disorders. The questionnaire has good reliability and validity [20]. Its internal reliability for the current study was 0.89.

#### 2.3.4. Time Organisation and Participation Scale

The TOPS [13] is a standardized self-reported scale to assess respondents’ difficulties in time organization while performing daily tasks. It is divided into four parts (35 items): (A) the pace of daily task performance (20 items) rated from 5 (*always*) to 1 (*never*); (B) how activities are organized throughout the day (five items) rated from 5 (*excellent*) to 1 (*very bad*); (C) the frequency of emotional responses following disorganization in time (eight items), rated from 5 (*never*) to 1 (*always*); and (D) two open-ended questions: (1) Do you have difficulty organizing your daily activities on time when a change occurs in a familiar routine? (2) Are you distracted by various stimuli and hence do not succeed in finishing your tasks on time? Respondents rate the frequency of the two-Part D items as 5 (*never*), 4 (*rarely*), 3 (*sometimes*), 2 (*usually*), or 1 (*always*). 

The item scores are averaged to calculate the TOPS total score in the Parts A through D domains; higher total scores indicate better daily functioning. The questionnaire has good reliability and validity [14]. Internal reliability for this study was 0.94. 

### 2.4. Data Analysis

The data were analyzed using IBM SPSS Statistics (ver. 26). The sample was normally distributed, and parametric testing was used. First, descriptive statistics were analyzed for each variable, and Pearson correlations were calculated among the variables. Next, the effects of cognitive-strategy use frequencies and moderating effects were examined using hierarchical multiple regression analyses [21]. When the interaction term is used to examine a moderation effect, multicollinearity between the predictors may result in underestimating regression coefficients or overestimating standard error values [22]. The Shapiro–Wilk statistic for dependent variable normality was 0.69 (*p* = 0.136). Linearity was found between the dependent and independent variables (0.187 < *r* < 0.554). For multicollinearity, the strongest intercorrelation was between gender and the CCSS (*r* = −0.165, *p* = 0.002). The minimum tolerance value was 0.948, the maximum Mahalanobis distance was 3.98, and the maximum Cook’s distance was 0.138. A scatterplot of residuals showed that most residuals were between −2 and +2.

The EF (DEX) and moderator (cognitive strategies) were mean-centered prior to statistical analysis to address this potential problem. The effect on the criterion variable (daily functioning) was examined after the predictor and moderator variables were entered as independent variables in Stage 1. The interaction term between the predictor and moderator variable was entered in Stage 2 as the independent variable.

## 3. Results

### 3.1. Data Analysis

A total of 336 students (180 women, 156 men) aged 18 to 40 years from universities across Israel—44 (13.1%) from southern, 62 (18.5%) from northern, and 230 (68.5%) from central regions—completed the questionnaires. Table 1 presents their demographic characteristics.

### 3.2. Frequency of Cognitive Strategies

The most frequently used compensatory cognitive strategies (according to the CCSS) were *prioritizing a “to-do” list* to aid time management and organization (65.2% of participants reported using this strategy *often* to *all the time*) and *using reminder functions* on computers or personal electronic devices such as smartphones or tablets (64% reported using this strategy *often* to *all the time*). The least frequently used strategies were *requesting accommodations* as needed, for example, *extra time* to complete assignments (73.5% reported using this strategy *never* to *rarely*) and *using a digital recorder* to make and later review short reminder memos (73.5% reported using this strategy *never* to *rarely*). Table 2 presents the frequency of cognitive strategy results.

### 3.3. Correlations between Cognitive Strategies, Executive Function, and Daily Functioning

Table 3 presents the descriptive statistics and correlations among study variables. The results indicated a significantly strong correlation between the DEX and TOPS scores (*r* = 0.51, *p* = 0.000). The other significant correlations were weak (ranging from −0.18 to −0.16).

### 3.4. Executive Function Effect on Daily Functioning: Moderating Effect of Cognitive Strategies

A hierarchical regression analysis was conducted to determine whether cognitive strategy use moderated the relationships between EF and daily functioning. The age and gender were entered in the first step and the DEX and TOPS mean scores in the second step. Table 4 presents the results, which indicate that the DEX significantly predicted daily functioning as assessed by the TOPS. 

**Figure 2:**
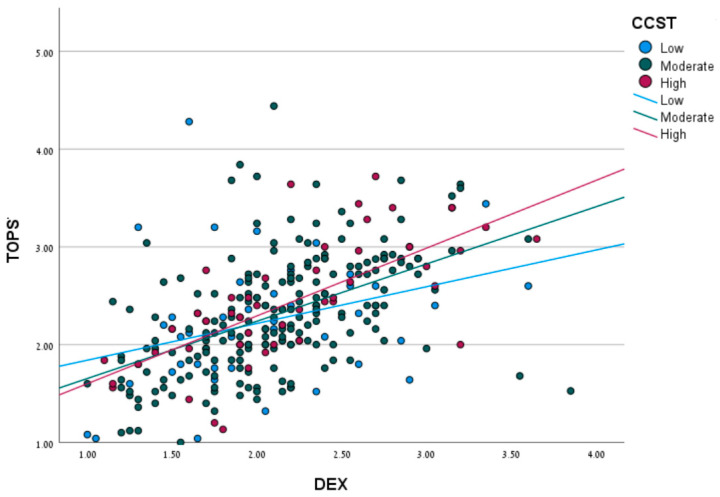
Moderation effect of cognitive strategy use on the relationships between executive functions and daily functioning.

Linear regression predicting the TOPS scores by DEX, CCSS, and DEX * CCSS interaction was used to assess the moderation relationship. Figure 2 demonstrates the moderation effect of cognitive strategy use (as assessed by CCSS) on the relationships between EF (by DEX) and daily functioning (by TOPS). Results showed a significant interaction (*B* = 0.01, *SE B* = 0.001, *p* = 0.07, 95% CI [0.01,0.02]). 

To probe that interaction, the relationship between the DEX and TOPS was calculated for three CCSS levels: −1.0 *SD*, −1.0 to 1.0 *SD*, and 1.0 *SD* from average. The results showed that the higher CCSS is, the stronger the association between the DEX and TOPS becomes. Specifically, a relatively low relationship was found between the DEX and TOPS (*B* = 0.47, *SE B* = 0.08, *p* < 0.001, 95% CI [0.31, 0.62]) for individuals who scored low on the CCSS; a relatively moderate relationship (*B* = 0.56, *SE B* = 0.06, *p* < 0.001, 95% CI [0.45, 0.67]) for individuals scoring moderate on the CCSS; and a relatively high relationship (*B* = 0.65, *SE B* = 0.07, *p* < 0.001, 95% CI [0.51, 0.79]) for individuals scoring high.

## 4. Discussion

The present research investigated how EF, daily functioning, and cognitive strategy use interact to gain a deeper theoretical and clinical understanding. Furthermore, the present study’s results highlight the significance of using cognitive strategies to enhance these relationships. This study found that the use of cognitive strategies moderates this relationship, showing that their frequent use strengthens the relationships and thus contributes to better daily functioning.

This study’s frequency-of-use results support previous studies that used the CCSS questionnaire [1,18]. The current study results indicate that some strategies are found with high frequency among the research participants. Examples include (No. 1) reminder functions on a computer or personal electronic device (e.g., smartphone, tablet) and (No. 20) prioritizing “to-do” lists to aid time management and organization. It also shows that the participants used other strategies with very low frequency, such as (No. 15) logging off email periodically to limit distractions and (No. 18) using a digital recorder to make and later review short reminder memos. 

The literature is replete with lists and descriptions of cognitive-strategy types (e.g., [2,23,24]. By looking at the most (above the average of 2.66) and least frequent strategies reported by research participants, it is possible to divide and characterize the strategies in the current study according to the accepted divisions in the literature. These three classifications address (a) external and internal strategies [6,25,26], (b) learning or self-regulation strategies, and (c) strategies directed at the task, environment, or person [2].

This study’s most frequent strategies can be typed as internal, and the less frequent as external. *Internal strategies* are those not apparent to others, such as (No. 9) performing one task at a time rather than multitasking and (No. 11) minimizing the number of open tabs/windows when on the Internet. *External strategies* are those apparent to another person, such as (No. 13) requesting accommodations as needed and (No. 16) requesting agenda or meeting notes. 

The most frequent strategies in this study also can be typed as self-regulation, and the less frequent as learning strategies. *Self-regulation strategies* require checking, monitoring, planning, predicting, and gauging performance, such as (No. 3) organizing to accomplish the most challenging task at the best time of day and (No. 8) rechecking work after a break. *Learning strategies* include acquiring, selecting, organizing, or rehearsing material to be learned, such as (No. 17) asking others to minimize interruptions when working and (No. 19) slowing down to avoid situations that require fast reaction time. 

Finally, cognitive strategies can be directed at the task, environment, or person. *Environment* or *task-oriented strategies* involve analyzing or modifying external demands or adjusting one’s attention to a specific task or environmental feature. *Person-oriented strategies* enhance the person’s retention, comprehension, and general abilities to attend, monitor, and regulate performance [23]. In this study, the most frequent strategies were directed toward the person. Examples include (No. 10) establishing a routine of putting “key” items in the same place in the house or workspace and (No.14) using personal electronic devices to record written documents. The less frequent strategies were directed toward the task or environment, such as (No. 5) slowing the pace of communication in meetings and (No. 6) confirming what others say by repeating the “gist” back to them.

Although this discussion of the strategy division is only descriptive, it distinguishes the more common from the less common strategies. This study’s participants report more frequent use of internal strategies, which enable self-regulation and are directed at the person.

### 4.1. Correlations between Cognitive Strategies, EF and Daily Functioning

The results reveal a highly significant correlation between EF as assessed by the DEX and daily functioning as assessed by the TOPS and significant weak correlations between EF and daily functioning to using cognitive strategies. Theoretical models and research findings previously connected daily functional deficits with deficient EF (e.g., [27,28] and support the correlations found in this study. 

Planning and organizing time for daily activities is an important prerequisite for managing daily life [29]. Proper time organization enables a sense of control and satisfaction; it reduces feelings of stress, leading to participation that is more meaningful [30,31]. Effective time organization and its related EF are key to executing daily activities and to independence [32,33]. 

Whereas most prior studies addressed the relationships between cognitive strategies and learning and achievement [34], this study examines cognitive strategies and daily functioning as assessed by the TOPS. Very few studies dealt with this connection. Sturkenboom et al. [35] found compromised efficiency of applied cognitive-strategy behaviors in a certain pattern of people with Parkinson’s disease that declines with disease progression. Meyer [36] described how cognitive strategies support adolescents with celiac disease self-management or with less effective management abilities. 

The correlation in this study between EF as assessed by the DEX and cognitive strategies was found to be significant but weak. As such, the weak relationship may imply that the EF relationship with strategies is *indirect* and that increased strategy use could strengthen the *direct* relationship between EF and daily functioning.

### 4.2. Executive Function Effect on Daily Functioning: Moderating Effect of Cognitive Strategies 

All individuals use strategies to manage the performance of occupations undertaken in daily life, regardless of whether they are aware of these strategies [2]. As the study results demonstrate, a strategy can be more than an intervention’s goal; it can extend beyond compensatory methods. However, using strategies daily plays a vital role in learning and in performing tasks [37,38]. The contribution of cognitive strategies to the relationship between EF and daily functioning can be explained from a theoretical viewpoint through cognitive load theory (CLT) and self-regulated learning (SRL) and from a clinical viewpoint through therapeutic interventions in which daily functioning improves by using cognitive strategies.

When discussing how cognitive strategies moderate the relationships between EF and daily functioning, CLT [39] and SRL [40] can help explain the moderation effect. The CLT posits that, because of working memory’s limited facilities, understanding effective teaching and learning revolves around the amount of information a learner is asked to process and how that information is presented [41]. *Cognitive load* commonly describes the mental-resource level demanded from the human brain for a specific task [42] and closely correlates with the person’s performance. A task whose demands are optimally matched to a user will keep the cognitive load at an appropriate level, leading to good user performance with high learning efficiency [39,43].

A crucial aspect of strategy use is reducing demands on working memory. For instance, students with dyslexia often struggle with working memory overload and insufficient processing space for new information [44]. Students who use strategies to support EF processes can effectively recognize their weaknesses and strengths [45,46] and can directly influence their academic performance [46,47]. Strategies may also support EF to manage the cognitive load better, concentrate on one topic at a time, and remain goal-oriented [46,48]. 

Within SRL, “learners set goals for their learning and then attempt to monitor, regulate and control their cognition, motivation and behavior, guided and constrained by their goals and the contextual features of the environment” [49] (p. 453). Knowledge and application of strategies play a significant role in the SRL process. It is key to a student’s ability to organize better the learning environment (time, place, material), plan learning activities, set goals, choose appropriate strategies to achieve goals, self-monitor the learning process, self-evaluate the learning outcome, and make appropriate adjustments [45,46]. Self-regulation embodies a crucial part of academic performance and cognitive development in student learning because it involves the student’s control over selecting and applying cognitive strategies [50,51].

The CLT and SRL theories complement each other and help explain the significant contribution of frequent cognitive-strategy use to the relationship between EF and daily functioning.

The literature highlights the importance, from a clinical viewpoint, of using strategies to promote cognitive functional performance. Within the rehabilitation context, the term *strategy* often is linked with compensation and viewed as the product of intervention [52,53,54]. Evidence-based reviews and meta-analyses of research studies consistently demonstrated that intervention approaches focused on strategies produce the best outcomes across groups. These groups have included persons with a learning disability [55,56], brain injury [5,57,58], stroke [59,60], Parkinson’s disease [61], and schizophrenia [62]. 

Huckans et al. [63] found that, following cognitive-strategy training treatment, participants reported significantly increased use of compensatory cognitive strategies and day planners; increased perception that these strategies were useful to them; increased life satisfaction; and decreased depressive, memory, and cognitive symptom severity. Kaizerman-Dinerman et al. [64] found that among people with schizophrenia, the intervention group had the larger number of cognitive strategies, better efficiency, more correct answers, and presented a significant increase in the use of four cognitive strategies (rereading, talking aloud, fixing appointments, and covering lines).

That evidence supports the contribution of cognitive-strategy use in identifying clinical populations. However, to the author’s knowledge, this study is the first to spotlight the strengthening effect of using cognitive strategies on the relationship. Only a few studies have dealt with the moderation effect of strategies. For example, Menéndez-Espina et al. [65] found that women acquire a greater variety of coping strategies that moderate between job insecurity and mental health. In contrast, men have only one, social withdrawal, which is also significant in the female group. Leone et al. [15] examined the effects of maladaptive cognitive–emotion regulation strategies on the relationship between alcohol use problems and negative relationship-conflict behaviors. 

Costley [50] found that cognitive-strategy use moderates the EF–daily functioning relationship, demonstrating that it can help overcome unclear instruction and produce higher levels of student learning. This study highlights the value of applying cognitive strategies in general and specifically in relationships with extraneous cognitive loads. It shows that cognitive strategies have a positive relationship with germane cognitive loads, illustrating the value of promoted cognitive strategies in online instructional situations and that they mitigate the extraneous load’s harmful effects on the germane load. Many students may struggle to learn when extraneous-load levels are high; this study suggests that applying cognitive learning strategies may help them overcome some difficulties.

### 4.3. Limitations and Future Research

This study referred to strategies in general, which is its main limitation—specifically, the reference to strategies was to only their frequency of use. The literature showed that strategies should be examined beyond their frequency, referring to their effectiveness and the degree of mental effort invested in implementing them [26]. Further, additional elements (e.g., demographic variables, awareness of strategy use, contexts in which strategies are used, simultaneous use of several strategies, and control over their effectiveness) might enable or hinder the frequency of using strategies. A follow-up study examining strategies for the frequency of use, effectiveness, and effort to implement may be very effective for understanding the elements underlying their use. 

Additionally, there was no attribution in this study to the students who reported disabilities. Follow-up studies should divide the population into groups and examine differences between the groups in daily functioning, EF, and strategy use. Finally, this research is based on students’ self-reported EF, daily functioning, and cognitive strategies. Future research should look at more concrete, objective measures of student outcomes, such as a controlled experiment wherein students perform a daily task with the frequency of using cognitive strategies observed. 

## 5. Conclusions

University students experience significant cognitive demands in their coursework, which could lead to mental and behavioral health problems (e.g., depression or alcohol use disorders) [66] that may adversely affect cognitive functioning [67]. 

This study’s results have practical implications on two levels. First, they can raise awareness among students of common strategies students reported in this and previous studies. Exposure to multiple strategies increases the chance that students will use them to promote their daily functioning. Second, the results can be used to map strategies as part of intervention processes. This is especially meaningful for students who are challenged to manage their day-to-day functioning and balance occupation, daily functioning, work, and leisure.

These clinicians might consider possible intervention modes that include addressing mental health problems, recommending academic accommodations, or providing psychoeducation on compensatory cognitive strategies to support academic performance. An instrument that quantifies current strategy use would benefit clinicians in educating patients on compensatory cognitive strategies as part of the interventions. Such an instrument would allow the clinicians to gauge whether psychoeducation was necessary (i.e., determine whether the student currently uses strategies in their everyday life) and track changes in the strategy used throughout the intervention [1]. 

## Figures and Tables

**Table 1:** Participants’ demographic characteristics.

Demographic	Mean (±SD)
Age (years)	24.61 (2.62)
	Frequency (%)
Gender	
Men	156 (46.4)
Women	180 (53.6)
Family status	
Single	275 (81.8)
Married without children	29 (8.6)
Married with children	27 (8.0)
Other (divorced, widowed)	5 (1.5)
Field of study	
Medicine	18 (5.4)
Therapy	61 (18.2)
Education	32 (9.5)
Computers	44 (13.1)
Engineering	68 (20.2)
Social science	18 (5.4)
Law	17 (5.1)
Architecture	4 (1.2)
Communication	16 (4.8)
Economic	29 (8.6)
Life science	19 (5.7)
Humanities	10 (3.0)
Grade range (% score)	
90–100	112 (33.3)
80–90	144 (42.9)
70–80	64 (19.0)
60–70	15 (4.5)
50–60	1 (0.3)
Employed	
Yes	214 (63.7)
No	122 (36.3)
Previous first degree	
Yes	9 (2.7)
No	327 (97.3)
Disability	
Motor	4 (1.2)
Mental	21 (6.3)
Cognitive	2 (0.6)
Communication	2 (0.6)
Neurodevelopmental (ADHD, DCD, SLD)	56 (16.7)
Not specified	12 (3.6)
No diagnosis	239 (71.1)

**Table 2:** Descriptive statistics, self-reported engagement in compensatory cognitive strategies: Compensatory Cognitive Strategies Scale frequency and means.

	Strategy		Frequency (%)				
		Mean (±SD)	Never	Rarely	Sometimes	Often	All the Time
1	Reminder functions on computer or personal electronic device (e.g., smartphone, tablet)	3.77 (1.21)	17 (5.1)	44 (13.1)	60 (17.9)	94 (28.0)	121 (36.0)
2	Break down complex tasks into smaller steps	2.94 (1.05)	33 (9.8)	75 (22.3)	128 (38.1)	79 (23.5)	21 (6.3)
3	Organize to do most challenging tasks at best (i.e., most energetic) time of day	3.02 (1.15)	41 (12.2)	68 (20.2)	97 (28.9)	103 (30.7)	27 (8.0)
4	Minimize workspace distractions by closing the door, etc.	3.40 (1.17)	24 (7.1)	56 (16.7)	81 (24.1)	113 (33.6)	62 (18.5)
5	Slow pace of communication in meetings	2.00 (1.14)	152 (45.2)	87 (25.9)	50 (14.9)	38 (11.3)	9 (2.7)
6	Confirm what others say by repeating the “gist” back to them	2.50 (1.11)	81 (24.1)	82 (24.4)	106 (31.5)	59 (17.6)	8 (2.4)
7	Take notes while listening/viewing presentations to organize/reinforce information	2.45 (1.36)	119 (35.4)	65 (19.3)	64 (19.0)	58 (17.3)	30 (8.9)
8	Recheck work after a break	3.16 (1.28)	47 (14.0)	56 (16.7)	84 (25.0)	93 (27.7)	56 (16.7)
9	Do one thing at a time rather than multitask	3.12 (1.21)	44 (13.1)	51 (15.2)	108 (31.1)	88 (26.2)	45 (13.4)
10	Establish routine of putting “key” items in the same place in the house or workspace	3.10 (1.55)	91 (27.1)	29 (8.6)	58 (17.3)	72 (21.4)	86 (25.6)
11	Minimize number of open tabs/windows when on the Internet	2.92 (1.46)	88 (26.2)	45 (13.4)	73 (21.7)	66 (19.6)	64 (19.0)
12	Write out notes (in advance) to help verbally relay a sequence of events	1.94 (1.16)	179 (53.3)	49 (14.6)	68 (20.2)	30 (8.9)	10 (3.0)
13	Request accommodations as needed (e.g., extra time to complete assignments)	1.84 (1.15)	190 (56.5)	57 (17.0)	54 (16.1)	22 (6.5)	13 (3.9)
14	Use personal electronic devices to record written documents	3.24 (1.34)	54 (16.1)	40 (11.9)	81 (24.1)	92 (27.4)	69 (20.5)
15	Log off email periodically to limit distractions	1.77 (1.17)	211 (62.8)	46 (13.7)	40 (11.9)	24 (7.1)	15 (4.5)
16	Request agenda/meeting notes	2.41 (1.46)	127 (37.8)	54 (16.1)	73 (21.7)	55 (16.4)	27 (8.0)
17	Ask others to minimize interruptions when working	2.33 (1.31)	127 (37.8)	66 (19.6)	74 (22.0)	42 (12.5)	27 (8.0)
18	Use digital recorder to make/later review short reminder memos	1.77 (1.16)	215 (64.0)	32 (9.5)	51 (15.2)	27 (8.0)	11 (3.3)
19	Slow down; avoid situations that require fast reaction time	1.82 (1.04)	181 (53.9)	68 (20.2)	59 (17.6)	23 (6.8)	5 (1.5)
20	Prioritize “to-do” list to aid time management and organization	3.76 (1.29)	33 (9.8)	24 (7.1)	60 (17.9)	91 (27.1)	128 (38.1)

**Table 3:** Means, standard deviations, and correlations among study variables.

Variable	Mean	(±SD)	1	2	3	4
Age	24.61	2.62				
Gender			0.16 **			
Compensatory Cognitive Strategy Scale	2.66	0.57	0.05	−0.16 **		
Dysexecutive Questionnaire	3.85	2.12	0.00	−0.09	0.13 *	
Time Organization and Participation Scale	4.44	2.31	−0.00	−0.18 **	0.12 *	0.51 ***

*Note*. * *p* < 0.05, ** *p* < 0.01, *** *p* < 0.001.

**Table 4:** Stepwise regression: Predicting TOPS.

Variable	*B*	*SE B*	ß	*B*	*SE B*	ß
	Step 1			Step 2		
Age	0.00	0.01	0.02	0.00	0.01	0.00
Gender	−0.22	0.07	−0.18	−0.15	0.06	−0.12
Dysexecutive Questionnaire				0.55	0.06	0.50
Compensatory Cognitive Strategy Scale				0.04	0.05	0.04

*Note*. Step 1: *R*^2^ = 0.03, Δ *R*^2^ = 0.03, *p* = 0.008; Step 2: *R*^2^ = 0.28, Δ *R*^2^ = 0.25, *p* = 0.000.

## Data Availability

The datasets generated and/or analyzed during the current study are not publicly available due to ethical restrictions but are available from the corresponding author on reasonable request.
